# Crystal structure of (*Z*)-3-[5-chloro-2-(prop-2-yn­yloxy)phen­yl]-3-hy­droxy-1-[4-(tri­fluoro­meth­yl)phen­yl]­prop-2-en-1-one

**DOI:** 10.1107/S2056989015012748

**Published:** 2015-07-08

**Authors:** Aarti Dalal, Ramesh C. Kamboj, Dinesh Kumar, Mahendra Kumar Sharma, Nagendran Selvarajan

**Affiliations:** a Department of Chemistry, Kurukshetra University, Kurukshetra 136 119, Haryana, India; b Department of Chemistry, Hindu College, University of Delhi, Delhi 110 007, India; c Department of Chemistry, Indian Institute of Technology, New Delhi 110 016, India

**Keywords:** crystal structure, hy­droxy enone, tautomerisation, hydrogen bonding, photo-isomerisation

## Abstract

The title compound, C_19_H_12_ClF_3_O_3_, obtained by the photochemical transformation of 2-[5-chloro-2-(prop-2-yn­yloxy)benzo­yl]-3-[4-(tri­fluoro­meth­yl)phen­yl]oxirane adopts a *Z* conformation with respect to the enolic C=C double bond. The dihedral angle between the benzene rings is 12.25 (16)° and an intra­molecular O—H⋯O hydrogen bond closes an *S*(6) ring. An intra­molecular C—H⋯O inter­action also leads to an *S*(6) ring. In the crystal, very weak C—H⋯O inter­actions and short Cl⋯Cl contacts [3.3221 (16) Å] are seen, as well as weak aromatic π–π stacking inter­actions [centroid–centroid separation = 3.879 (2) Å].

## Related literature   

For background to 1,3-diketones, see: Andrae *et al.* (1997 ▸); Crouse *et al.* (1989 ▸); Diana *et al.* (1978 ▸); Nishiyama *et al.* (2002 ▸); Sheikh *et al.* (2009 ▸, 2013 ▸); Tchertanov & Mouscadet (2007 ▸).
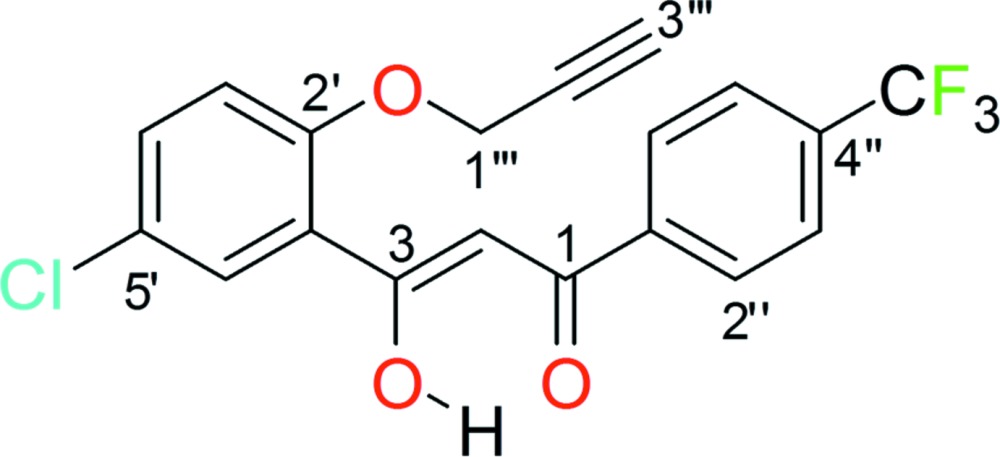


## Experimental   

### Crystal data  

C_19_H_12_ClF_3_O_3_
*M* *_r_* = 380.74
Triclinic, 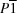

*a* = 8.2203 (12) Å
*b* = 9.3822 (14) Å
*c* = 12.3140 (18) Å
α = 90.150 (2)°
β = 109.201 (2)°
γ = 106.212 (2)°
*V* = 856.5 (2) Å^3^
*Z* = 2
Mo *K*α radiation
μ = 0.27 mm^−1^
*T* = 273 K
0.34 × 0.29 × 0.14 mm

### Data collection  

Bruker SMART CCD diffractometer
Absorption correction: multi-scan (*SADABS*; Bruker, 2000 ▸) *T*_min_ = 0.912, *T*_max_ = 0.962
8272 measured reflections
3016 independent reflections
2503 reflections with *I* > 2σ(*I*)
*R*_int_ = 0.022

### Refinement  

*R*[*F* ^2^ > 2σ(*F* ^2^)] = 0.068
*wR*(*F* ^2^) = 0.191
*S* = 1.10
3003 reflections
235 parameters
H atoms treated by a mixture of independent and constrained refinement
Δρ_max_ = 0.52 e Å^−3^
Δρ_min_ = −0.35 e Å^−3^

### 

Data collection: *SMART* (Bruker, 2000 ▸); cell refinement: *SAINT* (Bruker, 2000 ▸); data reduction: *SAINT*; program(s) used to solve structure: *SHELXS97* (Sheldrick, 2008 ▸); program(s) used to refine structure: *SHELXL97* (Sheldrick, 2008 ▸); molecular graphics: *SHELXTL* (Sheldrick, 2008 ▸); software used to prepare material for publication: *SHELXTL*.

## Supplementary Material

Crystal structure: contains datablock(s) I. DOI: 10.1107/S2056989015012748/hb7451sup1.cif

Structure factors: contains datablock(s) I. DOI: 10.1107/S2056989015012748/hb7451Isup2.hkl

Supporting information file. DOI: 10.1107/S2056989015012748/hb7451Isup3.cml

**Fig. 1.**
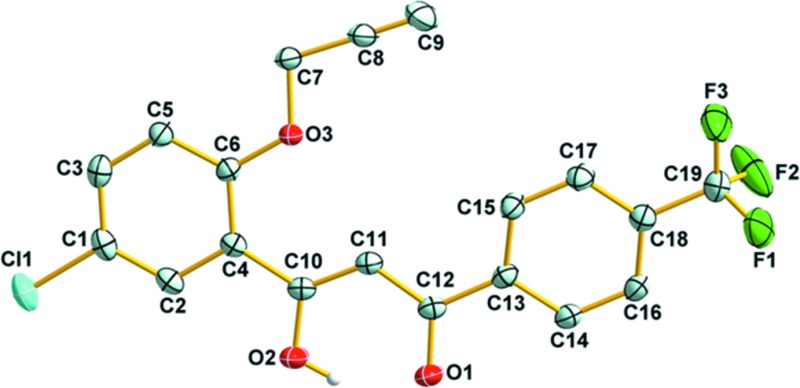
Molecular structure of the title compound showing displacement ellipsoids drawn at the 50% probability level.

**Fig. 2.**
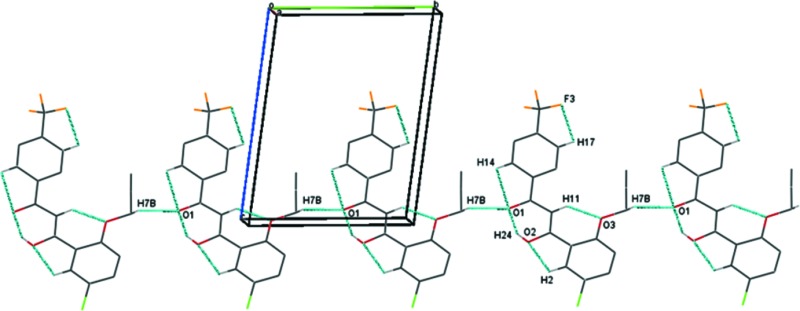
A view along the a axis of the inter- and intramolecular hydrogen bonds in the title compound (shown as dashed lines, see Table 1 for details). Hydrogen atoms not involved in hydrogen bonding are omitted for clarity.

**Fig. 3.**
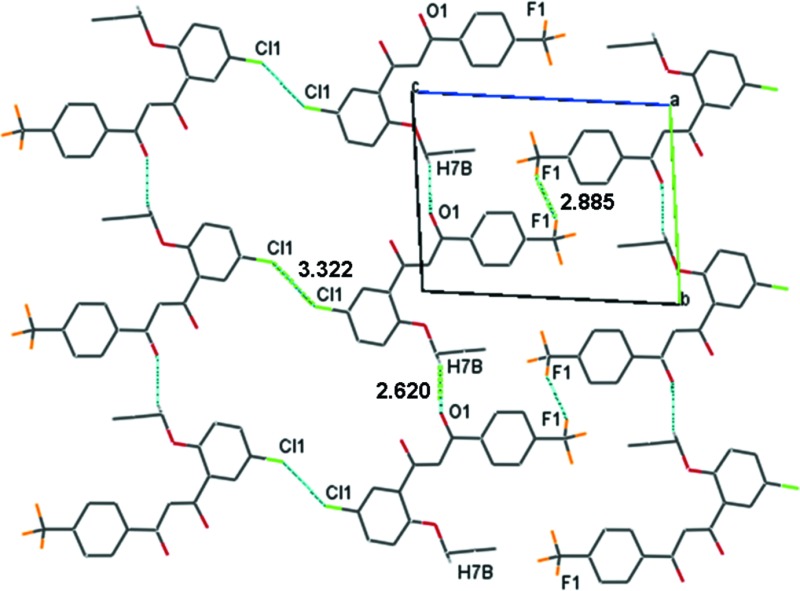
A view along the a axis showing F···F and Cl···Cl contact distances (dashed lines). Hydrogen atoms not involved in the interactions are excluded for clarity.

**Fig. 4.**
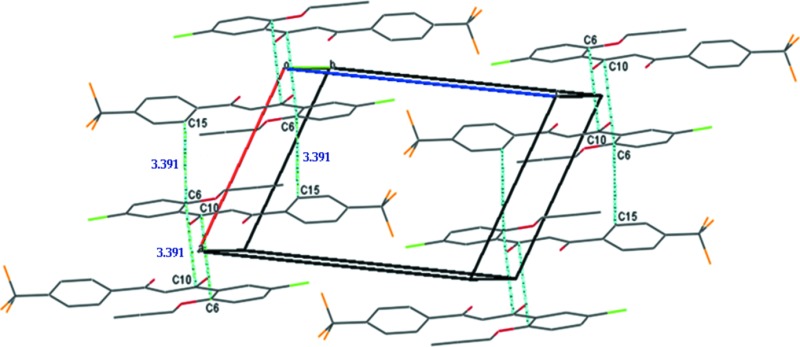
A partial view along b axis of the π–π interactions (dashed lines) in the crystal packing of the title compound. All hydrogen atoms are omitted for clarity.

. DOI: 10.1107/S2056989015012748/hb7451fig1.tif
Mol­ecular structure of the title compound showing displacement ellipsoids drawn at the 50% probability level.

a . DOI: 10.1107/S2056989015012748/hb7451fig2.tif
A view along the *a* axis of the inter- and intra­molecular hydrogen bonds in the title compound (shown as dashed lines, see Table 1 for details). Hydrogen atoms not involved in hydrogen bonding are omitted for clarity.

a . DOI: 10.1107/S2056989015012748/hb7451fig3.tif
A view along the *a* axis showing F⋯F and Cl⋯Cl contact distances (dashed lines). Hydrogen atoms not involved in the inter­actions are excluded for clarity.

b . DOI: 10.1107/S2056989015012748/hb7451fig4.tif
A partial view along *b* axis of the π–π inter­actions (dashed lines) in the crystal packing of the title compound. All hydrogen atoms are omitted for clarity.

CCDC reference: 1403411

Additional supporting information:  crystallographic information; 3D view; checkCIF report

## Figures and Tables

**Table 1:** Hydrogen-bond geometry (, )

*D*H*A*	*D*H	H*A*	*D* *A*	*D*H*A*
O2H24O1	0.82	1.79	2.529(3)	149
C11H11O3	0.93	2.09	2.747(3)	126
C7H7*B*O1^i^	0.97	2.62	3.476(4)	147

Symmetry code: (i) 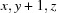.

## supplementary crystallographic information

### S1. Chemical context

The functionalized β-hy­droxy­enones (or 1,3-diketones) are medicinal significant compounds showing anti­viral (Diana *et al.*, 1978), insecticidal (Crouse *et al.*, 1989), anti-sunscreen (Andrae *et al.*, 1997), anti­oxidant (Nishiyama *et al.*, 2002), and more important HIV-1 Integrase (IN) inhibitor (Tchertanov & Mouscadet, 2007) activity. Also, 1,3-diketones are the building blocks for the synthesis of core heterocycles (Sheikh *et al.*, 2009), and biologically important metal complexes (Sheikh *et al.*, 2013). Our inter­est in the catalyst free photochemical organic transformation led us to synthesize the title compound and we report herein on its crystal structure (Fig. 1).

### S2. Synthesis and crystallization

A de­oxy­genated solution of 2-{5-chloro-2-(prop-2-ynyl­oxy)benzoyl}-3-{4-(tri­fluoro­methyl)­phenyl}­oxirane (2.0 mmol, 760 mg) in aceto­nitrile (100 ml) contained in a Pyrex glass vessel was purged with N_2_ for 30 min. and then irradiated under N_2_ atmosphere with light from a 125W Hg-vapor lamp for 90 min. The removal of solvent under reduced pressure yielded a gummy mass that was chromatographed over a silica gel column. The column was eluted with increasing proportion of ethyl acetate in pet ether-ethyl acetate mixture to obtain the desired photoproduct. In addition to this, another photoproduct 8-chloro-2-(4-tri­fluoro­methyl­phenyl) benzo[b]furo[2,3-e]oxepin-10(4*H*)-one was also separated from the photolysate. The resulting pale yellow solid was filtered, washed several times with pet ether and then dried in vacuo overnight to yield the desired (*Z*)-3-{5-chloro-2-(prop-2-ynyl­oxy)phenyl}-1-{4-(tri­fluoro­methyl)­phenyl}-3-hy­droxy­prop-2-en-1-one, (267 mg, 35% yield). The pale yellow re­cta­ngular crystals, suitable for X-ray structure analysis, were obtained by recrystallization from ethanol by slow evaporation at room temperature after several days (m.p. 383-385 K).

FT—IR (KBr) *ν*: 3418 (-OH), 2150 (C≡C), 1713 (C=O), 1605 (C=C) cm^-1^; ^1^H NMR (400 MHz, CDCl_3_) *δ*, ppm: 8.08 (2H, d, *J* = 8.2 Hz, H-3'', 5''), 7.95 (1H, d, *J* = 2.7 Hz, H-6'), 7.73 (2H, d, *J* = 8.3 Hz, H-2'', 6''), 7.44 (1H, dd, *J* = 8.8, 2.7 Hz, H-4'), 7.23(1H, s, H-2), 7.03 (1H, d, *J* = 8.8 Hz, H-3'), 4.82 (2H, d, *J* = 2.4 Hz, OCH_2_-1'''), 2.63 (1H, t, *J* = 2.3 Hz, ≡CH-3'''); ^13^C NMR (100 MHz, CDCl_3_):*δ*, ppm: 57.00, 99.21, 114.67, 122.34, 125.05, 125.63,126.58, 127.40, 127.62, 130.30, 132.84, 133.54, 133.86, 138.72, 154.99, 183.24, 183.69.

### S3. Refinement

Crystal data, data collection and structure refinement details are summarized in Table 2. All non-hydrogen atoms were refined anisotropically. The positions of the hydrogen atoms were fixed according to a riding model and were refined isotropically.

### Crystal data

**Table:**

C_19_H_12_ClF_3_O_3_	*Z* = 2
*M**_r_* = 380.74	*F*(000) = 388.0
Triclinic, *P*1	*D*_x_ = 1.476 Mg m^−^^3^
Hall symbol: -P 1	Mo *K*α radiation, λ = 0.71073 Å
*a* = 8.2203 (12) Å	Cell parameters from 3035 reflections
*b* = 9.3822 (14) Å	θ = 2.3–25.6°
*c* = 12.3140 (18) Å	µ = 0.27 mm^−^^1^
α = 90.150 (2)°	*T* = 273 K
β = 109.201 (2)°	Block, colorless
γ = 106.212 (2)°	0.34 × 0.29 × 0.14 mm
*V* = 856.5 (2) Å^3^	

### Data collection

**Table:**

Bruker SMART CCD diffractometer	3016 independent reflections
Radiation source: fine-focus sealed tube	2503 reflections with *I* > 2σ(*I*)
Graphite monochromator	*R*_int_ = 0.022
ω scans	θ_max_ = 25.0°, θ_min_ = 1.8°
Absorption correction: multi-scan (*SADABS*; Bruker, 2000)	*h* = −9→9
*T*_min_ = 0.912, *T*_max_ = 0.962	*k* = −11→11
8272 measured reflections	*l* = −14→14

### Refinement

**Table:**

Refinement on *F*^2^	Primary atom site location: structure-invariant direct methods
Least-squares matrix: full	Secondary atom site location: difference Fourier map
*R*[*F*^2^ > 2σ(*F*^2^)] = 0.068	Hydrogen site location: inferred from neighbouring sites
*wR*(*F*^2^) = 0.191	H atoms treated by a mixture of independent and constrained refinement
*S* = 1.10	*w* = 1/[σ^2^(*F*_o_^2^) + (0.0963*P*)^2^ + 0.390*P*] where *P* = (*F*_o_^2^ + 2*F*_c_^2^)/3
3003 reflections	(Δ/σ)_max_ = 0.001
235 parameters	Δρ_max_ = 0.52 e Å^−^^3^
0 restraints	Δρ_min_ = −0.35 e Å^−^^3^

### Special details

**Table:**

Geometry. All e.s.d.'s (except the e.s.d. in the dihedral angle between two l.s. planes) are estimated using the full covariance matrix. The cell e.s.d.'s are taken into account individually in the estimation of e.s.d.'s in distances, angles and torsion angles; correlations between e.s.d.'s in cell parameters are only used when they are defined by crystal symmetry. An approximate (isotropic) treatment of cell e.s.d.'s is used for estimating e.s.d.'s involving l.s. planes.
Refinement. Refinement of *F*^2^ against ALL reflections. The weighted *R*-factor *wR* and goodness of fit *S* are based on *F*^2^, conventional *R*-factors *R* are based on *F*, with *F* set to zero for negative *F*^2^. The threshold expression of *F*^2^ > σ(*F*^2^) is used only for calculating *R*-factors(gt) *etc*. and is not relevant to the choice of reflections for refinement. *R*-factors based on *F*^2^ are statistically about twice as large as those based on *F*, and *R*- factors based on ALL data will be even larger.

### Fractional atomic coordinates and isotropic or equivalent isotropic displacement parameters (Å^2^)

**Table:**

	*x*	*y*	*z*	*U*_iso_*/*U*_eq_	
Cl1	0.10694 (14)	1.10578 (13)	1.42182 (7)	0.0913 (4)	
O3	0.2888 (3)	1.16996 (19)	0.99885 (16)	0.0600 (5)	
O2	0.1279 (3)	0.7541 (2)	1.1146 (2)	0.0766 (7)	
H24	0.1237	0.6813	1.0756	0.115*	
C13	0.2457 (4)	0.7250 (3)	0.8037 (2)	0.0508 (6)	
C12	0.2108 (4)	0.7295 (3)	0.9149 (2)	0.0525 (7)	
C4	0.1949 (3)	1.0149 (3)	1.1317 (2)	0.0474 (6)	
C2	0.1511 (4)	1.0020 (3)	1.2318 (2)	0.0558 (7)	
H2	0.1147	0.9080	1.2557	0.067*	
C6	0.2471 (3)	1.1588 (3)	1.0975 (2)	0.0485 (6)	
C10	0.1852 (3)	0.8758 (3)	1.0691 (2)	0.0493 (6)	
C11	0.2284 (4)	0.8668 (3)	0.9711 (2)	0.0531 (7)	
H11	0.2707	0.9545	0.9408	0.064*	
C15	0.3271 (4)	0.8532 (3)	0.7619 (3)	0.0585 (7)	
H15	0.3643	0.9449	0.8054	0.070*	
C18	0.3000 (4)	0.7117 (3)	0.5930 (2)	0.0575 (7)	
C5	0.2558 (4)	1.2823 (3)	1.1638 (3)	0.0602 (7)	
H5	0.2906	1.3771	1.1410	0.072*	
C17	0.3537 (4)	0.8470 (3)	0.6579 (3)	0.0618 (7)	
H17	0.4077	0.9339	0.6313	0.074*	
C1	0.1610 (4)	1.1259 (4)	1.2959 (2)	0.0620 (8)	
F3	0.4185 (4)	0.8295 (3)	0.4541 (2)	0.1194 (9)	
C7	0.3400 (4)	1.3122 (3)	0.9602 (3)	0.0611 (7)	
H7A	0.4485	1.3760	1.0179	0.073*	
H7B	0.2448	1.3590	0.9473	0.073*	
C14	0.1936 (4)	0.5899 (3)	0.7376 (3)	0.0633 (8)	
H14	0.1400	0.5026	0.7641	0.076*	
C3	0.2136 (4)	1.2658 (4)	1.2624 (3)	0.0656 (8)	
H3	0.2204	1.3491	1.3067	0.079*	
C8	0.3725 (5)	1.2911 (4)	0.8531 (3)	0.0750 (9)	
C16	0.2202 (5)	0.5826 (3)	0.6329 (3)	0.0686 (8)	
H16	0.1844	0.4911	0.5893	0.082*	
C19	0.3237 (5)	0.7041 (4)	0.4781 (3)	0.0763 (9)	
C9	0.3999 (8)	1.2763 (5)	0.7678 (4)	0.1171 (17)	
H9	0.4219	1.2645	0.6994	0.140*	
F2	0.1707 (4)	0.6729 (5)	0.3933 (2)	0.1649 (15)	
F1	0.4002 (7)	0.6064 (4)	0.4652 (3)	0.1871 (19)	
O1	0.1593 (3)	0.6070 (2)	0.9541 (2)	0.0759 (7)	

### Atomic displacement parameters (Å^2^)

**Table:**

	*U*^11^	*U*^22^	*U*^33^	*U*^12^	*U*^13^	*U*^23^
Cl1	0.0980 (7)	0.1272 (9)	0.0637 (6)	0.0341 (6)	0.0464 (5)	0.0084 (5)
O3	0.0908 (14)	0.0395 (10)	0.0603 (12)	0.0176 (9)	0.0411 (11)	0.0111 (8)
O2	0.1181 (19)	0.0490 (12)	0.0843 (15)	0.0281 (12)	0.0595 (14)	0.0234 (10)
C13	0.0497 (14)	0.0436 (14)	0.0584 (16)	0.0184 (11)	0.0138 (12)	0.0050 (12)
C12	0.0546 (15)	0.0452 (14)	0.0606 (16)	0.0198 (12)	0.0194 (13)	0.0107 (12)
C4	0.0428 (13)	0.0516 (15)	0.0478 (14)	0.0153 (11)	0.0147 (11)	0.0084 (11)
C2	0.0511 (15)	0.0652 (17)	0.0518 (15)	0.0155 (13)	0.0199 (12)	0.0142 (13)
C6	0.0479 (14)	0.0473 (14)	0.0502 (14)	0.0137 (11)	0.0168 (11)	0.0048 (11)
C10	0.0491 (14)	0.0440 (14)	0.0553 (15)	0.0151 (11)	0.0173 (12)	0.0134 (11)
C11	0.0618 (16)	0.0408 (14)	0.0579 (16)	0.0137 (12)	0.0235 (13)	0.0104 (12)
C15	0.0625 (17)	0.0463 (15)	0.0667 (18)	0.0101 (13)	0.0272 (14)	−0.0019 (13)
C18	0.0556 (16)	0.0593 (17)	0.0560 (16)	0.0204 (13)	0.0145 (13)	0.0020 (13)
C5	0.0659 (18)	0.0521 (16)	0.0641 (17)	0.0166 (13)	0.0252 (15)	0.0005 (13)
C17	0.0632 (17)	0.0519 (16)	0.0696 (19)	0.0092 (13)	0.0284 (15)	0.0047 (14)
C1	0.0552 (16)	0.084 (2)	0.0492 (15)	0.0215 (15)	0.0204 (13)	0.0054 (14)
F3	0.152 (2)	0.1049 (18)	0.1022 (17)	−0.0047 (16)	0.0797 (17)	−0.0060 (14)
C7	0.0781 (19)	0.0425 (14)	0.0730 (19)	0.0208 (13)	0.0367 (16)	0.0161 (13)
C14	0.082 (2)	0.0418 (15)	0.0623 (18)	0.0163 (14)	0.0211 (15)	0.0068 (13)
C3	0.0672 (18)	0.071 (2)	0.0613 (18)	0.0240 (15)	0.0219 (15)	−0.0085 (15)
C8	0.109 (3)	0.0563 (18)	0.085 (2)	0.0361 (18)	0.057 (2)	0.0315 (16)
C16	0.091 (2)	0.0459 (16)	0.0621 (18)	0.0197 (15)	0.0182 (16)	−0.0030 (13)
C19	0.094 (3)	0.067 (2)	0.062 (2)	0.0193 (19)	0.0233 (19)	−0.0047 (16)
C9	0.196 (5)	0.104 (3)	0.111 (3)	0.073 (3)	0.105 (4)	0.051 (3)
F2	0.118 (2)	0.248 (4)	0.0599 (14)	−0.032 (2)	0.0136 (14)	0.0125 (18)
F1	0.372 (6)	0.176 (3)	0.126 (2)	0.180 (4)	0.148 (3)	0.048 (2)
O1	0.1138 (18)	0.0424 (11)	0.0871 (15)	0.0259 (11)	0.0525 (14)	0.0177 (10)

### Geometric parameters (Å, º)

**Table:**

Cl1—C1	1.745 (3)	C18—C17	1.377 (4)
O3—C6	1.364 (3)	C18—C16	1.382 (4)
O3—C7	1.416 (3)	C18—C19	1.494 (4)
O2—C10	1.310 (3)	C5—C3	1.366 (4)
O2—H24	0.8200	C5—H5	0.9300
C13—C14	1.384 (4)	C17—H17	0.9300
C13—C15	1.393 (4)	C1—C3	1.372 (5)
C13—C12	1.491 (4)	F3—C19	1.312 (4)
C12—O1	1.265 (3)	C7—C8	1.452 (4)
C12—C11	1.409 (4)	C7—H7A	0.9700
C4—C2	1.390 (4)	C7—H7B	0.9700
C4—C6	1.408 (4)	C14—C16	1.381 (4)
C4—C10	1.484 (4)	C14—H14	0.9300
C2—C1	1.371 (4)	C3—H3	0.9300
C2—H2	0.9300	C8—C9	1.161 (5)
C6—C5	1.388 (4)	C16—H16	0.9300
C10—C11	1.373 (4)	C19—F1	1.283 (4)
C11—H11	0.9300	C19—F2	1.299 (4)
C15—C17	1.372 (4)	C9—H9	0.9300
C15—H15	0.9300		
			
C6—O3—C7	119.4 (2)	C6—C5—H5	119.7
C10—O2—H24	109.5	C15—C17—C18	119.8 (3)
C14—C13—C15	117.9 (3)	C15—C17—H17	120.1
C14—C13—C12	119.7 (3)	C18—C17—H17	120.1
C15—C13—C12	122.4 (2)	C2—C1—C3	120.8 (3)
O1—C12—C11	121.7 (3)	C2—C1—Cl1	119.7 (3)
O1—C12—C13	118.0 (2)	C3—C1—Cl1	119.5 (2)
C11—C12—C13	120.2 (2)	O3—C7—C8	107.8 (2)
C2—C4—C6	117.9 (2)	O3—C7—H7A	110.1
C2—C4—C10	117.5 (2)	C8—C7—H7A	110.1
C6—C4—C10	124.6 (2)	O3—C7—H7B	110.1
C1—C2—C4	120.9 (3)	C8—C7—H7B	110.1
C1—C2—H2	119.6	H7A—C7—H7B	108.5
C4—C2—H2	119.6	C16—C14—C13	121.0 (3)
O3—C6—C5	122.6 (2)	C16—C14—H14	119.5
O3—C6—C4	117.4 (2)	C13—C14—H14	119.5
C5—C6—C4	120.0 (3)	C5—C3—C1	119.8 (3)
O2—C10—C11	120.1 (2)	C5—C3—H3	120.1
O2—C10—C4	114.1 (2)	C1—C3—H3	120.1
C11—C10—C4	125.9 (2)	C9—C8—C7	179.0 (4)
C10—C11—C12	122.4 (2)	C14—C16—C18	119.9 (3)
C10—C11—H11	118.8	C14—C16—H16	120.1
C12—C11—H11	118.8	C18—C16—H16	120.1
C17—C15—C13	121.4 (3)	F1—C19—F2	106.7 (4)
C17—C15—H15	119.3	F1—C19—F3	105.4 (4)
C13—C15—H15	119.3	F2—C19—F3	103.3 (3)
C17—C18—C16	120.0 (3)	F1—C19—C18	113.8 (3)
C17—C18—C19	120.2 (3)	F2—C19—C18	111.9 (3)
C16—C18—C19	119.8 (3)	F3—C19—C18	114.9 (3)
C3—C5—C6	120.6 (3)	C8—C9—H9	180.0
C3—C5—H5	119.7		
			
C14—C13—C12—O1	10.5 (4)	C4—C6—C5—C3	−0.2 (4)
C15—C13—C12—O1	−170.5 (3)	C13—C15—C17—C18	−0.4 (5)
C14—C13—C12—C11	−167.0 (3)	C16—C18—C17—C15	−0.2 (4)
C15—C13—C12—C11	12.0 (4)	C19—C18—C17—C15	178.4 (3)
C6—C4—C2—C1	−0.9 (4)	C4—C2—C1—C3	0.3 (4)
C10—C4—C2—C1	178.5 (2)	C4—C2—C1—Cl1	−179.5 (2)
C7—O3—C6—C5	−1.2 (4)	C6—O3—C7—C8	−178.7 (3)
C7—O3—C6—C4	179.3 (2)	C15—C13—C14—C16	−0.6 (4)
C2—C4—C6—O3	−179.7 (2)	C12—C13—C14—C16	178.4 (3)
C10—C4—C6—O3	1.0 (4)	C6—C5—C3—C1	−0.4 (5)
C2—C4—C6—C5	0.8 (4)	C2—C1—C3—C5	0.4 (5)
C10—C4—C6—C5	−178.5 (3)	Cl1—C1—C3—C5	−179.9 (2)
C2—C4—C10—O2	2.7 (4)	O3—C7—C8—C9	−158 (26)
C6—C4—C10—O2	−178.0 (2)	C13—C14—C16—C18	0.1 (5)
C2—C4—C10—C11	−177.9 (3)	C17—C18—C16—C14	0.3 (5)
C6—C4—C10—C11	1.3 (4)	C19—C18—C16—C14	−178.2 (3)
O2—C10—C11—C12	1.0 (4)	C17—C18—C19—F1	130.3 (4)
C4—C10—C11—C12	−178.2 (2)	C16—C18—C19—F1	−51.1 (5)
O1—C12—C11—C10	−2.2 (4)	C17—C18—C19—F2	−108.6 (4)
C13—C12—C11—C10	175.2 (2)	C16—C18—C19—F2	69.9 (4)
C14—C13—C15—C17	0.7 (4)	C17—C18—C19—F3	8.7 (5)
C12—C13—C15—C17	−178.3 (3)	C16—C18—C19—F3	−172.7 (3)
O3—C6—C5—C3	−179.7 (3)		

### Hydrogen-bond geometry (Å, º)

**Table:**

*D*—H···*A*	*D*—H	H···*A*	*D*···*A*	*D*—H···*A*
O2—H24···O1	0.82	1.79	2.529 (3)	149
C11—H11···O3	0.93	2.09	2.747 (3)	126
C7—H7*B*···O1^i^	0.97	2.62	3.476 (4)	147

Symmetry code: (i) *x*, *y*+1, *z*.
