# Supplementary figures and images for: Crystal structure of (Z)-3-[5-chloro-2-(prop-2-yn­yloxy)phen­yl]-3-hy­droxy-1-[4-(tri­fluoro­meth­yl)phen­yl]­prop-2-en-1-one
Source: Acta Crystallogr E Crystallogr Commun. 2015 Jul 8;71(Pt 8):o556–7. doi: 10.1107/S2056989015012748 (PMC4571394; doi:10.1107/S2056989015012748)

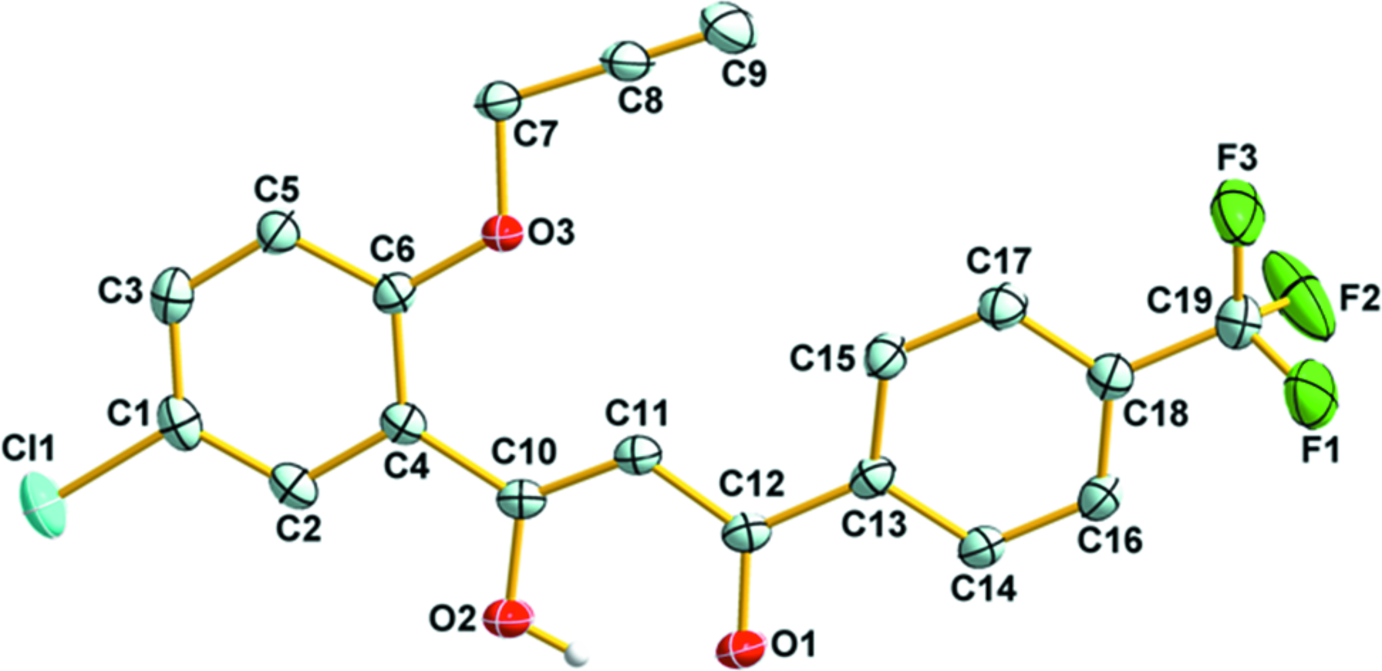

Supplement: Supplementary file 4 [file e-71-0o556-fig1.tif]

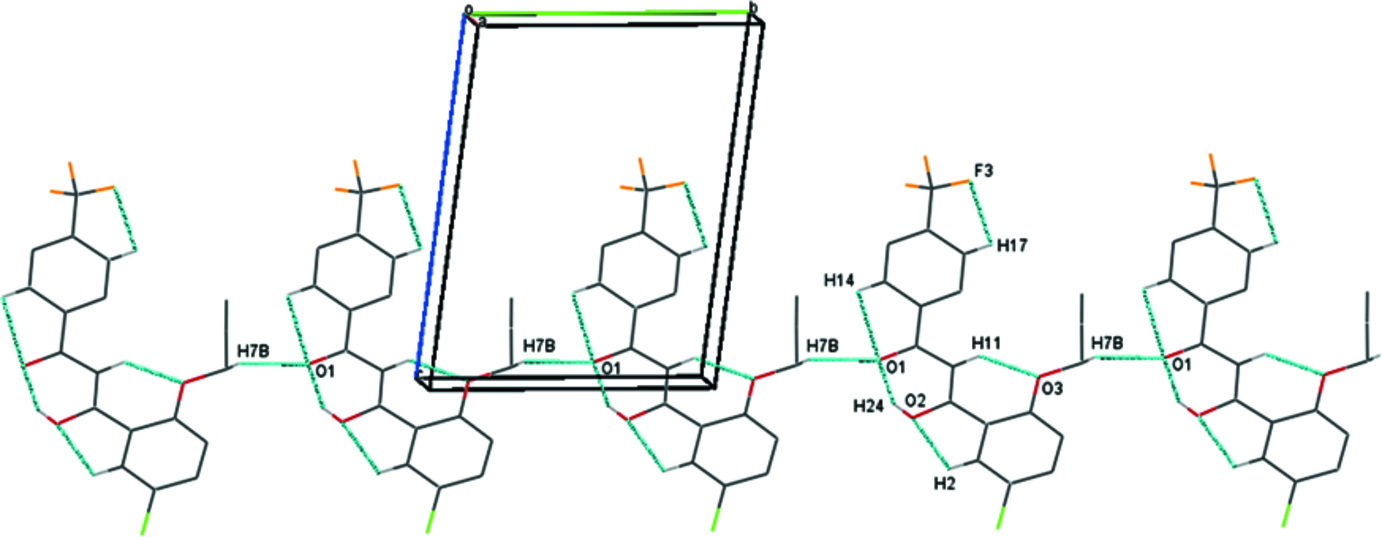

Supplement: Supplementary file 5 [file e-71-0o556-fig2.tif]

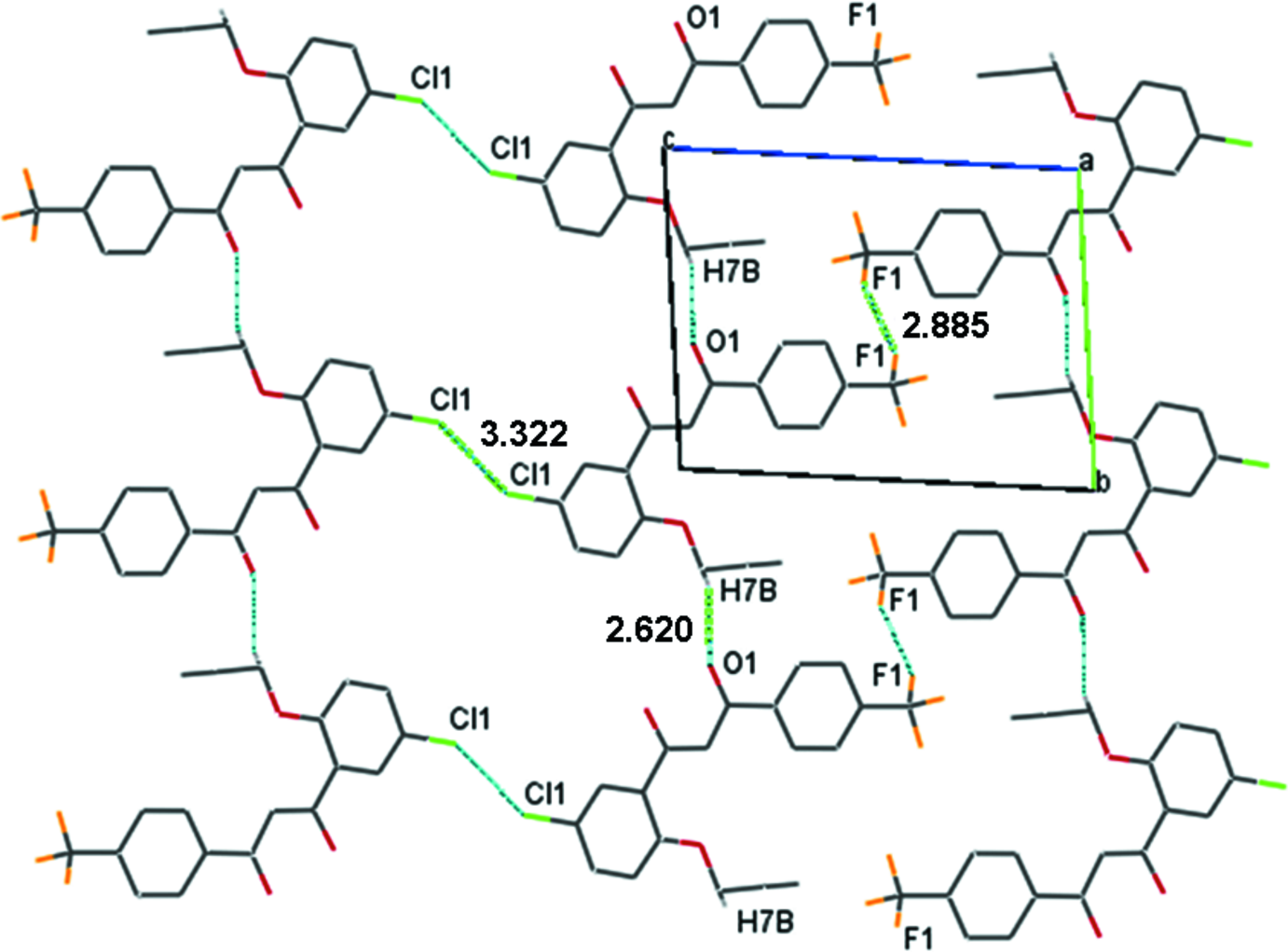

Supplement: Supplementary file 6 [file e-71-0o556-fig3.tif]

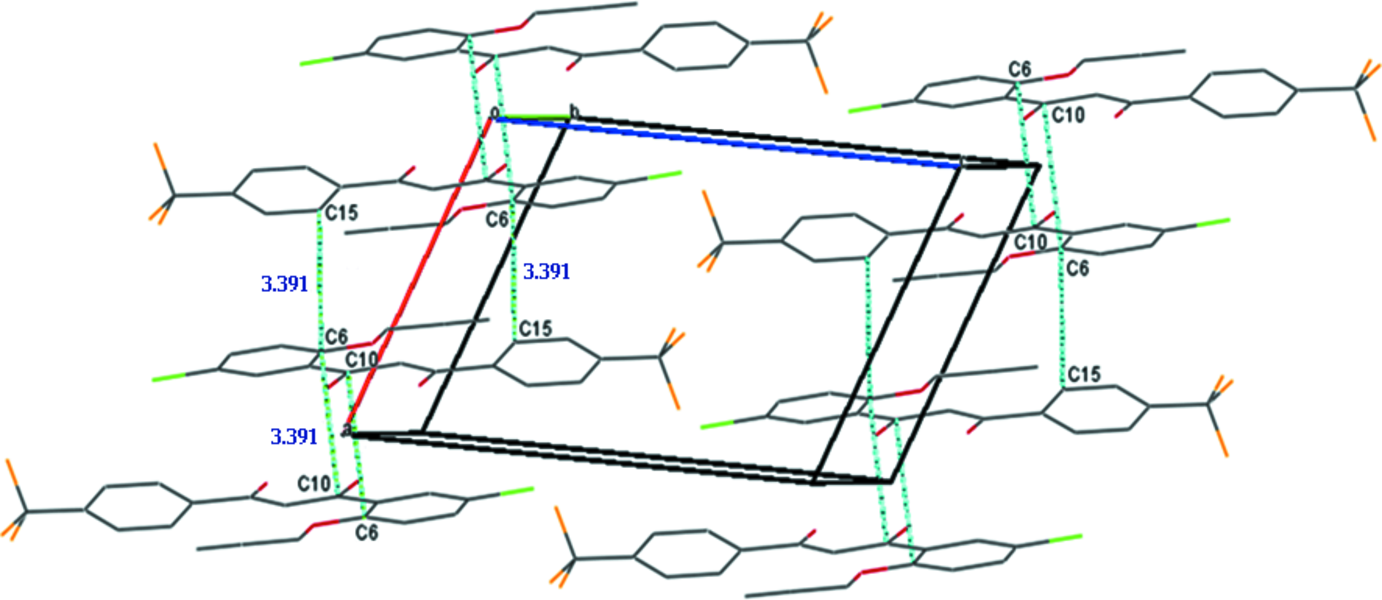

Supplement: Supplementary file 7 [file e-71-0o556-fig4.tif]
